# Characterization of defensome genes and mobile genetic Elements in different types of pasture soil agroecosystems from the Brazilian Amazon

**DOI:** 10.1007/s10123-026-00840-1

**Published:** 2026-05-19

**Authors:** Luan Filipe de Souza Pereira, Taynara Cristina Santos Tavares, David Tavares Martins, Carlos Willian Dias Dantas, Fernanda Olivia Rodrigues de Souza, Maria Clara Coelho Prazeres, Cristian Faturi, Hervé Louis Ghislain Rogez, Rommel Thiago Jucá Ramos, Oscar Victor Cardenas Alegria, Adriana Ribeiro Carneiro Nunes

**Affiliations:** 1 Center for the Valorization of Bioactive Compounds of the Amazon, Guamá, Science and Technology Park - Innovation Space, Federal University of Para, Belém, PA Brazil; 2 Laboratory of Bioinformatic and Genetic of Microorganisms, Pará, Center for High Performance Distributed Computing (CCAD), Federal University of Pará, Belém, 66075-110 Brazil; 3 Institute of Animal Health and Production, Federal Rural University of Amazonia, Belém, PA Brazil

**Keywords:** Bacteriophages, Host-Pathogen Interactions, Mobile genetic elements, Pasture, Defensome

## Abstract

The Amazon rainforest represents nearly 40% of the world’s tropical forests and has undergone extensive conversion to pasture, profoundly altering soil microbial communities. Given that bacteriophage-driven selective pressure shapes bacterial defense systems (the defensome) as well as mobile genetic elements (MGEs), we examined the diversity and distribution of these genetic components in native forest soils and in pasture soils under two management regimes (with and without fertilization) in the Brazilian Amazon. Metagenomic sequencing revealed pronounced differences in bacterial community structure between forest and pasture sites (*R* = 0.942), whereas phages communities exhibited no significant variation. Pasture soils—particularly those under fertilization—showed higher abundances of functional genes and mobile genetic elements, including conjugative plasmid-associated genes and insertion sequences. Defensome analyses indicated an increased prevalence of retrons and *Pycsar* systems in managed soils, while a greater diversity of defense genes was observed in non-fertilized pastures. A strong positive correlation was observed between defensome diversity and MGE diversity, suggesting coordinated dynamics between viral selective pressure and horizontal gene transfer. These findings indicate that forest-to-pasture conversion reshapes microbial functional potential and amplifies genetic mechanisms linked to phage defense and gene mobility, with potential consequences for ecosystem functioning and the dissemination of antimicrobial resistance.

**Supplementary Information:**

The online version contains supplementary material available at https://doi.org/10.1007/s10123-026-00840-1.

## Introduction

Soil microorganisms include bacteria, fungi, archaea, protozoa, and microalgae (Chen et al. 2024; Song et al. 2025), which play an essential role in the decomposition of organic matter, the cycling of elements, and the availability of nutrients for plants. This microbial activity holds a central role in soil processes, particularly due to the diversity, quantity, and functions developed by prokaryotes. In this sense, viruses are biological entities whose functions are poorly understood (Florent et al. 2022); however, they are central components of microbiomes, shaping the composition of microbial communities (Roux and Emerson 2022), and are essential for mediating microbial metabolism and functions in the soil (Tong et al. 2025). Furthermore, soils are recognized as major reservoirs of viral diversity, antibiotic resistance genes (ARGs) and antibiotic-resistant bacteria (ARB) (Shi et al. 2023; Roux and Emerson 2022). Bacteriophages, or phages, are viruses that infect bacteria and archaea, recognizing specific bacteria (Nie et al. 2024), and replicating within prokaryotic cells by exploiting their cellular machinery (Maffei et al. 2021; Mantynen et al. 2021; Chevallerau et al. 2022; Braga et al. 2020; Putzeys et al. 2024; Gummalla et al. 2023; Bloch and Wegrzyn 2024; García et al. 2023; Liang et al. 2025; Wegrzyn 2022). Phages are abundant across diverse ecosystems, including soils (Wu et al. 2023), and play a crucial role in shaping the dynamics and evolution of microbial communities (Shaidullina and Harms 2022; Mavrich and Hatfull 2017). They are known to have a limited rate of bacterial hosts (Torres-Barceló et al. 2025; Dunne et al. 2018). Metagenomic approaches have complemented studies of viral communities, showing their extensive genomic diversity in the environment of these domains (Roux and Emerson 2022).

In view of this, the selective pressure exerted by them contributes to the development of various bacterial defense mechanisms, defined as defensomes, related to the flow of genetic information disseminated by and against mobile genetic elements (MGEs - transposons, integrons, plasmids, and bacteriophages) through horizontal gene transfer (HGT) (Millman et al. 2022; Tan et al. 2025; Li et al. 2025; Almeida et al. 2025; Dorr et al. 2025). These mobile genetic elements are responsible for the capture, accumulation, and dissemination of resistance genes (Partridge et al. 2018). Many studies have proposed that mobile genetic elements, as well as anthropogenic impact and horizontal gene transfer, are driving factors for the introduction and dissemination of resistance mechanisms in microbial communities. Furthermore, MGEs are associated with more recent resistance mechanisms in untouched environments (Santos et al. 2022). These defense systems are divided into two large groups: innate or non-specific components, such as abortive infection systems, in which the cell initiates the process of programmed cell death to prevent the spread of viral infection (Lopatina et al. 2020); and adaptive components, the main example of which is the CRISPR-Cas system, which incorporates short sequences of MGEs, the spacers, that act as a molecular memory for future infections (Koonin and Makarova 2019; Beavogui et al. 2024).

The Amazon region encompasses an area of approximately 6 million km², representing about 40% of global tropical forests (Neto et al. 2019; Rosan et al. 2024). It is present in nine South American countries, concentrated mainly in Brazilian territory (De Oliveira Neves et al. 2024). Rural colonization in the Amazon has resulted in intense deforestation and, consequently, increased soil degradation, mainly due to the creation of pastures (Zenero et al. 2016). The conversion of forest to pasture stimulates the release of methane, a potent greenhouse gas, whose biotic cycle is driven by microorganisms (Kroeger et al. 2021).

Thus, these viruses are fluid entities, capable of merging and transforming through encounters. This can be called pluribiosis, a concept used to present the dynamism of viral agency, whose pathogenicity is contextual (Dupin 2023). Therefore, this work aims to characterize the genes related to the prokaryotic defensome and MGEs in different soils with different types of pasture and forest treatments.

## Materials and methods

### Study site

**Fig. 1:**
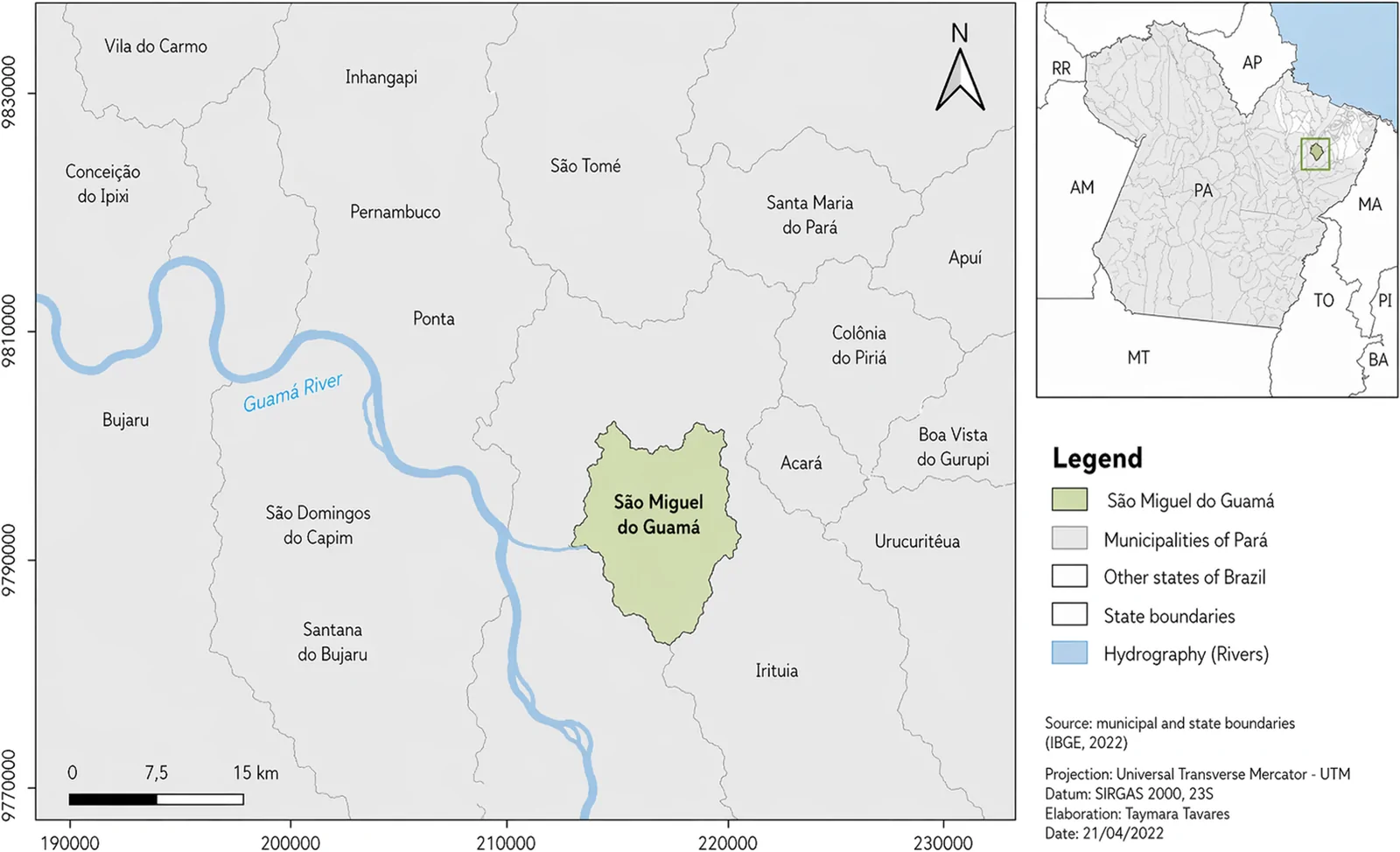
Location of the municipality of São Miguel do Guamá, Pará, Brazil

Soil samples were obtained in the municipality of São Miguel do Guamá, located in the northeastern region of the state of Pará, Brazil (Fig. 1). According to the Köppen classification, the area has a hot and humid climate, with an average annual temperature of 26 °C (Alvares et al. 2013). In the study area, approximately 82.1% of the soils correspond to Yellow Latosol, a type of medium-textured soil characterized by low natural fertility, high acidity, and strong leaching (Leite et al. 2024); these soils exhibit acidic characteristics.

The agroecosystems analyzed were characterized as native forest (F) (− 1.492534; −47.629718); pasture under treatment 1 (PT1), characterized by management with fertilizer application (limestone and chemical fertilizers) (− 1.507812; −47.646530); and pasture under treatment 2 (PT2), corresponding to management without the use of fertilizers (− 1.494170; −47.643097). The information about organic horizon, soil physicochemical characteristics and how the samples were collected are described in the study carried out by Tavares et al. (2025).

### Metagenomic data acquisition

The raw metagenomic data used in this study are currently available in the SRA/NCBI database under the access number: Forest (F1 - SRX27423983, F2 - SRX27423984, F3 - SRX27423985); PT1 (PT1.A - SRX27423980, PT1.B - SRX27423981, PT1.C - SRX27423982); PT2 (PT2.A - SRX27423986, PT2.B - SRX27423987, PT2.C - SRX27423988). It is important to note that the data employed here was generated through sequencing using the NextSeq 550 System High-Output Kit, for a read size of 2 × 150. However, the detailed methodology was described by Tavares et al. (2025).

### Metagenomic data processing and assembly

The quality of the sequences generated in triplicate was initially evaluated using FASTQC, version 0.11.9 (Saheb Kashaf et al. 2022). Subsequently, the data were subjected to trimming and filtering using Trimmomatic, adopting a minimum quality value of Phred 20 (Sewe et al. 2022).

Nevertheless, the previously processed sequences were assembled in MEGAHIT 2.4.3 (Li et al. 2015), using specific parameters for soil genomic data (meta-large mode). The generated contigs were evaluated for quality using MetaQuast 3.2 (Mikheenko et al. 2016).

### Bacterial diversity and functional profile and viral diversity

Taxonomic analyses of the assembled contigs were performed using the MicrobiomeAnalyst platform 2.0 (Lu et al. 2023). To evaluate the microbial community diversity within the samples, alpha diversity was assessed using the Simpson and Shannon indices and beta diversity was analysed via Analysis of Similarities (ANOSIM) and Non-metric Multidimensional Scaling (NMDS) (Dexter et al. 2018).

The results were evaluated for normality using the Shapiro-Wilk test, adopting a significance level of 5% (*p* < 0.05). The particle size distribution and alpha-diversity indices among the communities were subjected to Analysis of Variance (ANOVA) or the Kruskal-Wallis test, using Past 5.2 software 0. Subsequently, the graphs were generated in Python (Bouzenia et al. 2024), using the Matplotlib, Pandas, and Seaborn libraries.

For metagenome functional annotation, the assembled contigs were submitted to PROKKA (Seeman, 2014) to performs gene prediction (via Prodigal) of sequences. The Code Sequences (CDS) predicted were annotated via the Metagenomic Rapid Annotations using Subsystem Technology (MG-RAST) platform, version 4.0.3, which uses the SEED Subsystems database. The following parameters were used: e-value of 10⁻⁵, minimum identity of 80%, and length of 15 nucleotides (nt), for analysis of functional genes.

### Defensome, MGEs and prediction of host-virus interactions

Defensome-related genes were analyzed using the DefenseFinder v2.0.0 program (Tesson et al. 2024) with parameters of e-values ​​below 10⁻⁵ and minimum identity of 90%. Insertion sequences and transposon related genes were identified based on annotations obtained from PROKKA (Seemann 2014). Virus-host interactions were inferred using PhageScope (Wang et al. 2024). Viral and plasmid associated genes were identified using geNomad (Camargo et al. 2024).

### Results and discussions

The yield obtained from sequencing the different samples analyzed ranged from 20,050,310 to 38,965,502 reads, corresponding to the highest number of reads among the pasture sites. These metagenomic data were generated from a study project titled “Antimicrobial resistance profile of Amazonian soils” (Table 1).

**Table 1:** Metagenomic data obtained from sequencing

SRA accession number	Sample code	Number of reads	Number of bases	Size
SRR32075507	PT1A	38,965,502	11.7 G	4.5 Gb
SRR32075506	PT1B	29,138,757	8.8 G	3.4 Gb
SRR32075505	PT1C	28,698,973	8.6 G	3.3 Gb
SRR32075501	PT2A	20,050,310	6 G	2.3 Gb
SRR32075500	PT2B	23,589,666	7.1 G	2.8 Gb
SRR32075499	PT2C	22,360,409	6.7 G	2.7 Gb
SRR32075504	NFA	22,756,547	6.9 G	2.6 Gb
SRR32075503	NFB	20,956,929	6.3 G	2.4 Gb
SRR32075502	NFC	21,141,277	6.4 G	2.5 Gb

**Fig. 2:**
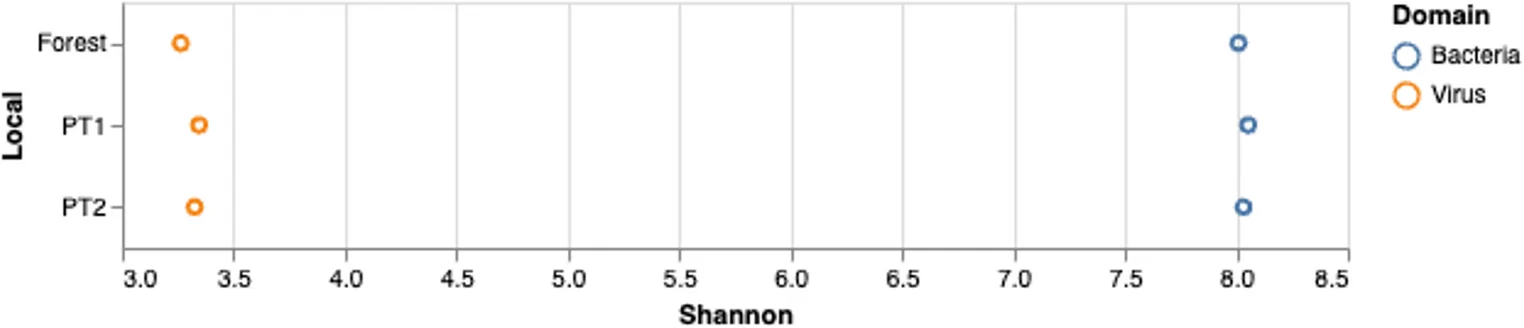
Shannon alpha diversity of different domains of bacteria and viruses in different study locations

In the analysis of alpha diversity using the Shannon index, high average values ​​were observed for the Bacterial domain among the pasture sites: 8.053 (± 0.027) in PT1, 8.04 (± 0.025) in PT2, and in the forest site: 7.994 (± 0.044). In the case of the Virus domain, the values ​​were lower in contrast to the previous one, with an average of 3.331 (± 0.032) in PT2, 3.306 (± 0.107) in PT1, and in the forest: 3.292 (± 0.084), without significant differences (Fig. 2).

Soil bacteria are important in nutrient cycling, plant growth, and plant adaptation to soil environmental stress (Costa et al. 2024; Alexandre 2025; Xu et al. 2024; Baker et al. 2024; Bacmaga et al. 2023). Viruses, on the other hand, play a key role in regulating bacterial communities by causing mortality in their hosts and altering community composition, modifying the genetic landscape and metabolic activities in ecosystems (Narr et al. 2017), having a significant impact on natural selection and influencing different bacterial species (Napflin et al. 2019).

The analysis of beta diversity for the bacterial domain showed strong data representation (NMDS; Stress = 0.032), revealing the difference in bacterial composition at the different sites (ANOSIM; *R* = 0.942; *p*-value = 0.009), as observed in the supplementary material, figure S1A. For the virus domain, acceptable data representation was observed (NMDS; stress = 0.171), but without differences between sites (ANOSIM; *R* = -0.136; *p*-value = 0.772) (supplementary material, figure S1B). However the absence of taxonomic differences in viral communities does not preclude functional differences in virus-host interactions, which may be an associated factor to the observed diversification of defense systems and mobile genetic elements. The study conducted by Rocha and Bikard (2022) has shown that different strains tend to carry very different repertoires of prophages, plasmids or transposable elements (Rocha and Bikard 2022). In a different study, Unzueta-Martínez and Girguis (2025) have shown that the genetic plasticity in a microbial community can result from constant selection by the host immune system, associating the diversity of genetic elements to a diversity of hosts as observed in this study.

Soil management in pasture systems can profoundly alter the soil microbiota, reshaping it into a community with a microbial composition distinct from that of forest soils, as observed within the bacterial domain (Khan et al. 2025). This finding corroborates previous studies showing that land-use changes drive shifts in specific microbial groups, thereby restructuring soil microbial communities (Burton et al. 2022; Venturini et al. 2022a, b, 2025). Such ecosystem modifications can also affect metabolic processes, including gas fluxes in soils, and may contribute to the disappearance of atmospheric methane sinks (Mir et al. 2023; Alvarez et al. 2023). Consequently, these alterations could serve as potential biomarkers for changes in microbial diversity and soil health. However, we recognize that stratified sampling (0–10 cm) could provide additional resolution.

### Bacterial and viral taxonomic composition

**Fig. 3:**
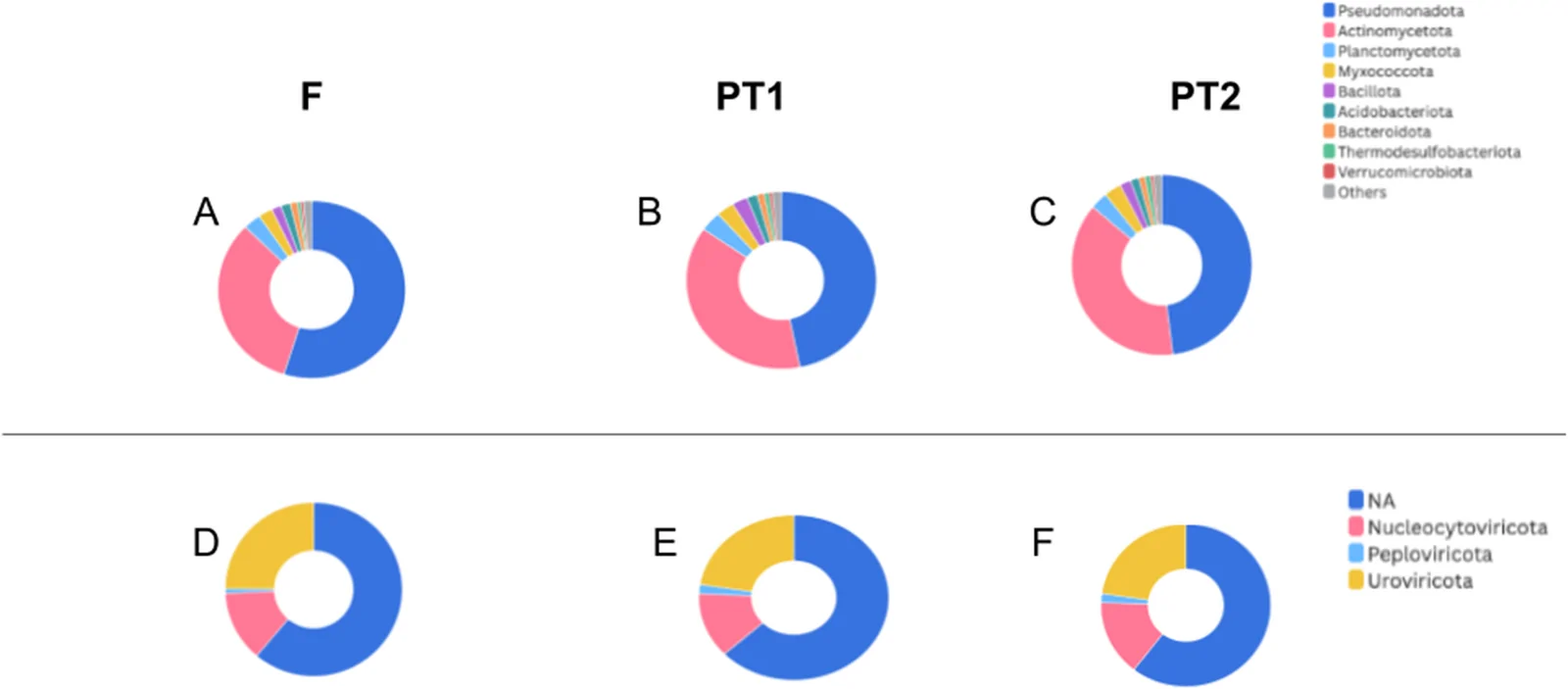
Composition of the microbial community by phylum in three sites: forest, pasture with treatment 1, and pasture with treatment 2 (**A-C**: bacteria; **D-F**: viruses)

The taxonomic composition of the bacterial domain in the three sites analyzed showed similarities across the three locations studied. The prominent groups at the phylum level were Pseudomonadota, followed by Actinomycetota (Fig. 3A-C). A similar situation was found for the viral domain, where the most relevant identified phyla were Uroviricota, followed by Nucleocytoviricota and Peploviricota, to a lesser extent (Fig. 3D-F).

The predominance of the phyla Pseudomonadota, Actinomycetota, and Uroviricota in pasture soils with and without fertilizer use has been observed in studies, indicating an increase in the Actinomycetota phylum and a decrease in Pseudomonadota in soils with fertilizer use (Lyu et al. 2025). This was also observed in this study (Fig. 3.B), where a greater abundance of the first phylum was described compared to forest soils, and a decrease in the second compared to pasture soils without fertilizer use. The Pseudomonadota phylum is relevant to human health, as is the case with species such as *Pseudomonas aeruginosa*, which is an opportunistic pathogen and has the ability to resist antibiotics (Pang et al. 2018). The phylum Actinomycetota is responsible for a large production of secondary metabolites with bioactive properties (Yang et al. 2023), which is of significant importance for biotechnological studies, in addition to helping in the cycling of soil organic carbon (Zhang et al. 2024).

Regarding the analysis of viruses, the phylum *Uroviricota* consists of bacteriophages that can infect a wide range of hosts present in the soil (Zhou et al. 2023) and could have relevance in ecosystems, which shows the importance of analyzing these types of components beyond the more predominant domain of bacteria.

Taxonomic composition at the family level showed proximity across different sites, with a greater emphasis on bacterial families typical of this type of ecosystem, such as *Nitrobacteraceae* and *Streptomycetaceae*, among others. The presence of families of clinical interest, such as Enterobacteriaceae (Potter et al. 2016), is also noteworthy among the different sites, although in PT2 they are grouped by the similarity of their composition at the family level (supplementary material figure S2A). The composition of virus families at the different sites showed a greater predominance of unassigned families, followed by the Mimiviridae and Poxviridae families, without a clustering of replicates among the sites studied (supplementary material figure S2B).

Sites PT1 and the forest were more similar in terms of the relative abundance of bacterial families, whereas PT2 exhibited greater diversity in relative abundance. This pattern corroborates previous studies showing that fragmented pastures, such as PT2—comparable to intensive pasture systems—tend to harbor higher diversity (Fajardo et al. 2025; Lasanta et al. 2024). The most abundant families identified were *Enterobacteriaceae* and *Streptomycetaceae*. Members of the *Enterobacteriaceae* family include numerous human pathogens commonly associated with antimicrobial resistance (Ma et al. 2023). In contrast, bacteria from the *Streptomycetaceae* family are widely studied for their ability to produce bioactive compounds with antimicrobial activity, as demonstrated by Chin et al. (2023), who isolated actinobacterial strains active against *Staphylococcus aureus*. These findings highlight the complexity of microbial community composition, the coexistence of distinct biogeochemical processes, and the selective pressures shaping community assembly. Alterations in these ecosystems may therefore influence the emergence of opportunistic pathogenic communities. In the case of abundant viral families identified, such as Mimiviridae, known for harboring “giant viruses,” they have double-stranded DNA (dsDNA) that infects a wide range of aquatic eukaryotes, such as phytoplankton (Claverie and Abergel 2018). However, although this family is predominant in aquatic environments, these viruses can also be found in forest soils and pasture soils (Venturini et al. 2022a, b). Nevertheless, it is worth noting the presence of Poxviridae, which contains viruses of interest to agriculture (Zhao et al. 2023), because these pasture sites are used for livestock.

### Bacterial functional genetic components

**Fig. 4:**
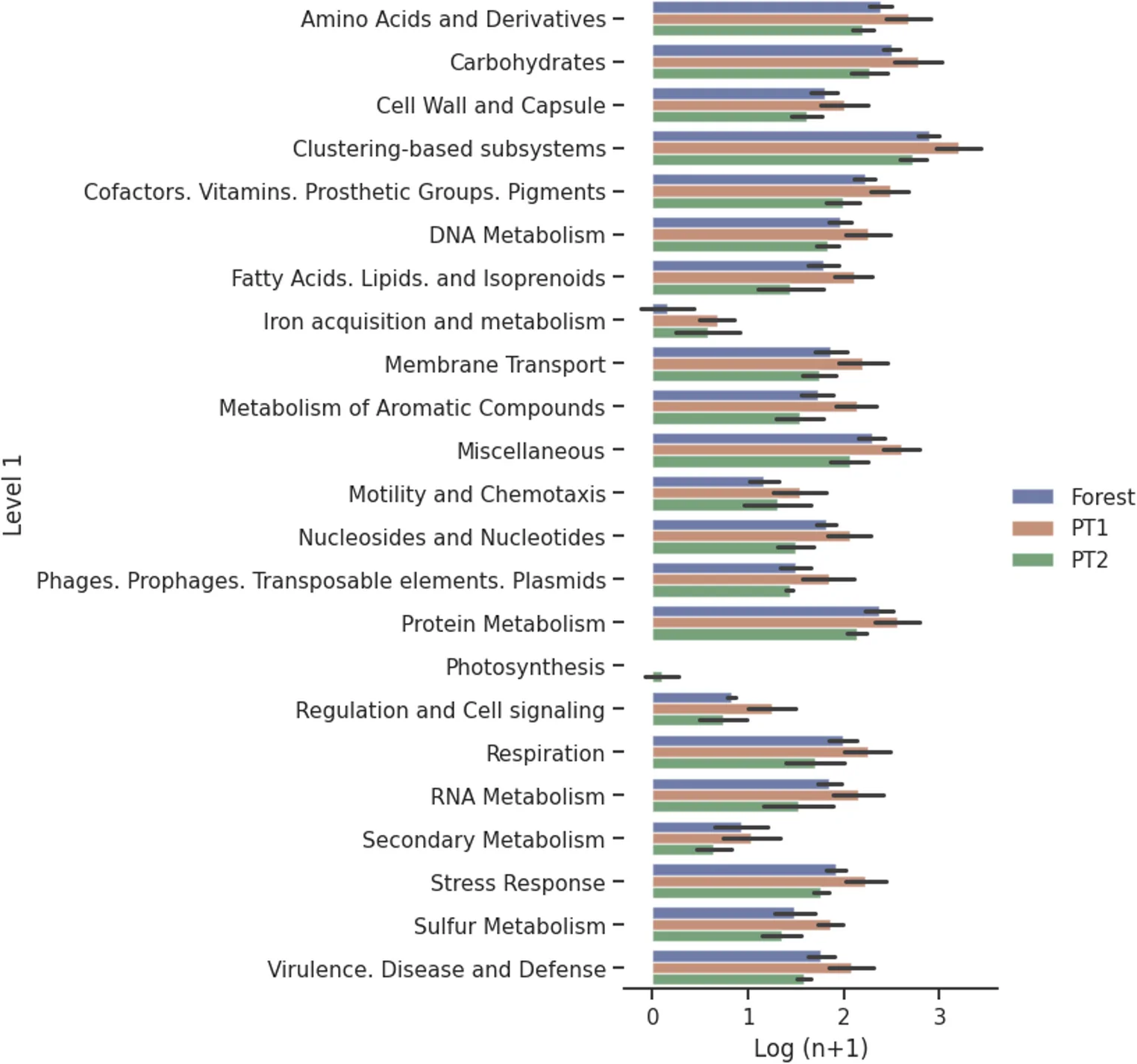
Classification of functional genes according by MG-RAST

Analysis of the distribution of functional genes in different sites showed that almost all different groups of functional genes are more abundant in the PT1 site, with significant differences (Kruskal-Wallis; chi^2^ = 22.07; *p*-value = 1.611 × 10^-5^), with differences between the PT1 and PT2 sites (Mann-Whitney; U = 1324; *p*-value = 6.887 × 10^-6^) and forest (Mann-Whitney; U = 1675; *p*-value = 0.002). At level 1, genes associated with cellular functional metabolism were prominent, alongside components involved in horizontal transfer processes, including phages, prophages, transposons, and plasmids (Fig. 4).

A high abundance of functional genes related to protein metabolism, among others, was observed at the PT1 site, possibly indicating enhanced physiological activity in bacteria compared to other locations. Similar functional gene profiles have frequently been reported in metagenomic analyses (He et al. 2018). In contrast, mobile genetic elements—primarily responsible for horizontal gene transfer—were predominant, including transposons, integrons, plasmids, and bacteriophages. These elements may contribute to essential functions in soil bacteria, such as iron acquisition and metabolism (Martins et al. 2017), underscoring their importance for sustaining microbial populations in these ecosystems.

### Defensome analysis

**Fig. 5:**
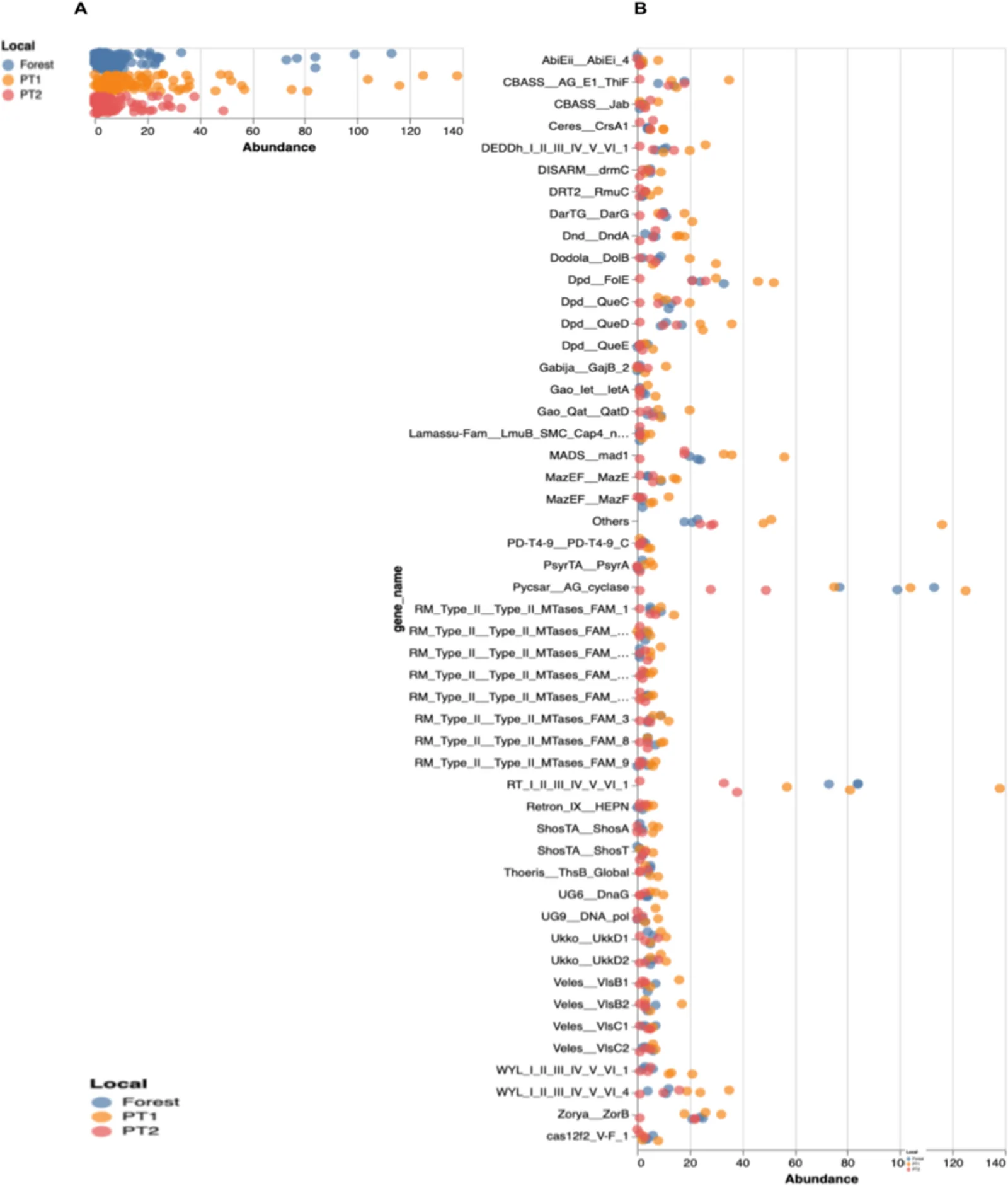
Distribution of genes related to the defensome (**A**) sites and (**B)** genes. A major abundance was observed in PT1

This analysis revealed that it was possible to observe a relative abundance of genes, especially at site PT1 (Fig. 5A), indicating a high frequency of specific genes. However, genetic diversity showed values ​​of 4,026 (± 0.62) at PT2, 3,732 (± 0.145) at PT1, and 3,236 (± 0.1) at the forest site, suggesting that, despite the high number of genes present at PT1, the greatest diversity of bacterial defense systems was in soil with management without fertilization (PT2).

Fertilization in PT1 could have favored the dominance of specific bacterial groups, resulting in a high density of restricted defense systems. In contrast, the greater gene diversity observed in PT2 could indicate that the absence of soil treatment with fertilizer would act as an inducer of bacterial communities adapted to the use of these compounds, forcing resident microbial communities to have different defense genes against genetic exchange. Recent studies corroborate what has been previously described about the complexity of the defensome, which is shaped by the heterogeneity of the environment and the density of virus communities in this type of ecosystem, so that soils with greater nutritional stress can harbor different taxa and, consequently, different defense mechanisms (Meaden et al. 2025; Huang et al. 2025; Lori et al. 2023; Xu et al. 2020).

Nevertheless, the presence of genes related to defense mechanisms against phages was mainly observed; among these, genes of different types stand out, such as retrons (RT) and Pycsar, see Fig. 5B.

The predominance of systems such as Retrons and Pycsar (pyrimidine cyclase system for antiphage resistance) (Patel et al. 2023) may reflect the complexity of bacterial immune responses in managed soils. As described in the literature, the Pycsar system employs nucleotides to activate immune signaling—a sophisticated mechanism that triggers multiple proteins throughout the cell to disrupt viral replication (Hobbs et al. 2024; Tal et al. 2021; Hobbs et al. 2022). Fertilized soils (e.g., PT1) provide increased resources that can be exploited by microbial communities (Zhou et al. 2025). Pycsar mechanisms typically degrade or interfere with the formation of key cellular components, such as nucleic acids, membranes, or metabolites, thereby reducing viral replication kinetics at a high metabolic cost to the infected cell (Feltin et al. 2025; Athukoralage and White 2022). Thus, the high occurrence of this system in PT1 suggests that the greater resource availability at this site may support the substantial metabolic demands of pyrimidine cyclase–based immune signaling.

Additionally, the detection of retrons underscores the importance of mechanisms that protect bacterial populations against phages (Liu et al. 2024). The accumulation of retrotranscribed DNA (msDNA), produced through the reverse transcription cascade mediated by ribonuclease H, functions as a critical sensor for phage infection (Dai et al. 2025; Khan et al. 2024; Nakamura et al. 2025; Bobonis et al. 2022). The prominent presence of these molecular detectors in PT1 suggests an active co-evolutionary dynamic, in which bacteria invest in complex defense systems to mitigate the impact of the high viral pressure observed in Amazonian pasture soils.

### Analysis of the genetic components of mobile elements (MGE)

The diversity of genes related to mobile elements was similar in pasture sites with average genetic diversity values ​​of PT1 3.07 (± 0.064), PT2 3.052 (± 0.039) and in the forest of 2.924 (± 0.022), with significant differences (ANOVA; F = 5.143; *p*-value = 0.014), with the differences between forest sites and PT1 (Tukey’s test; Q = 5.588; *p*-value = 0.018) and PT2 (Tukey’s test; Q = 4.912; *p*-value = 0.03).

**Fig. 6:**
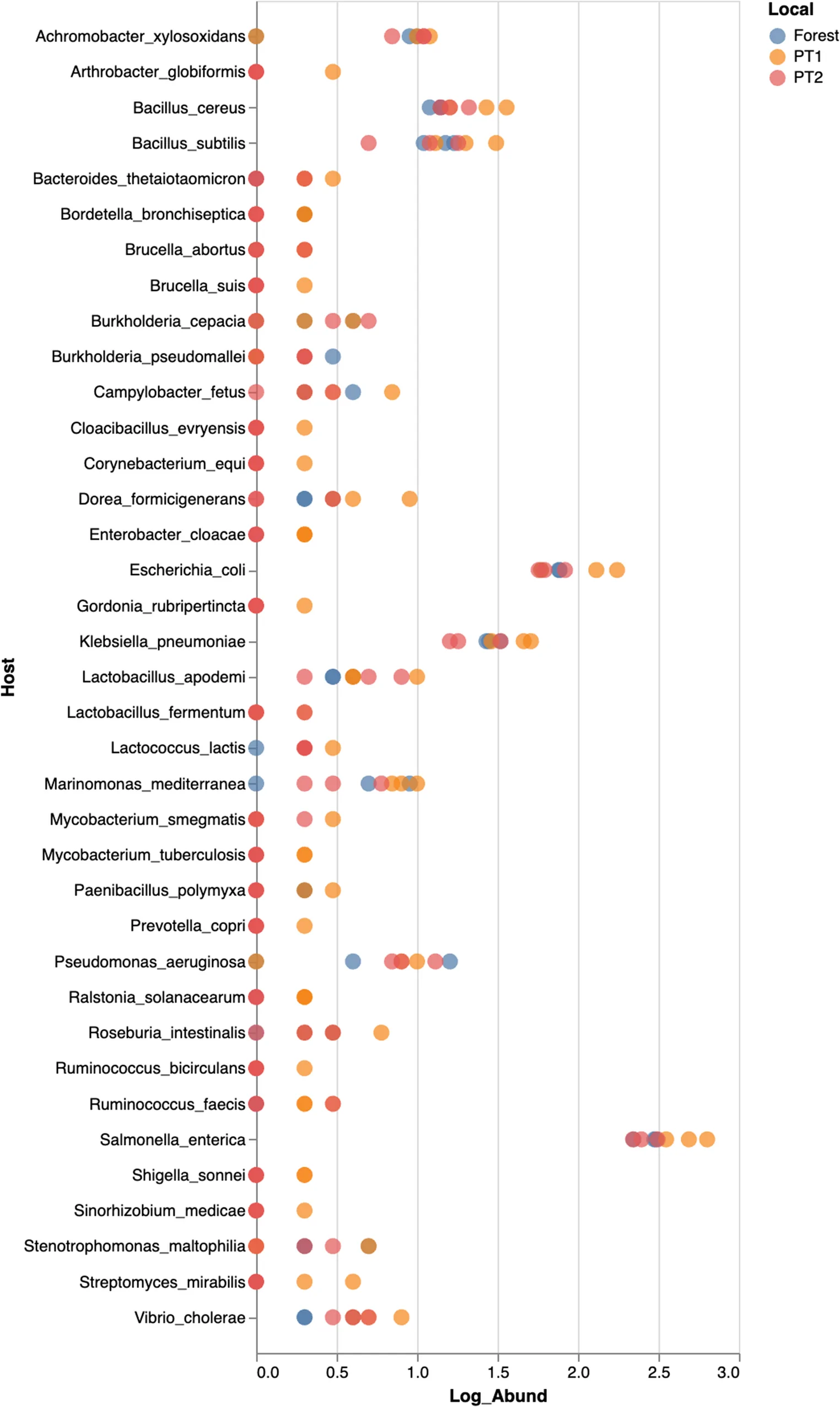
Determination of bacterial hosts of phages at different sites. It was possible to see a major prevalence of enteric bacteria

In turn, the analysis of the identified phage hosts highlights enteric bacteria at the PT2 and forest sites. The most prominent species were *Salmonella enterica*, *Escherichia coli*, *Klebsiella pneumoniae*, and also *Bacillus cereus* and *Bacillus subtilis* (Fig. 6).

The most abundant host bacteria were *Salmonella enterica* and *Escherichia coli*; they are opportunistic pathogens for higher organisms (Han et al. 2024; Gomes et al. 2016) and are frequently associated with gastrointestinal infections, with the ability to form biofilms and resistance to different antimicrobials (Sharma et al. 2024). Some studies, such as that of Kobakhidze et al. (2024), suggest genetic recombination by phages, allowing for wide possibilities of genetic material exchange, which contributes to their virulence and antimicrobial resistance. Therefore, continuous monitoring of soil microbiota and assessment of the possibility of dissemination of antimicrobial resistance genes in soils are essential, especially in pasture soils, which, in the long term, may contribute to the dissemination of these genes (Seyoum et al. 2023).

Among the genetic elements involved in the insertion of bacteriophage genetic components, the enzymatic components related to nucleases are most prominent at the PT1 site, in contrast to the PT2 site (supplementary material figure S3).

Pasture soils differed from forest soils in the abundance of several mobile genetic elements (DNA polymerase exonuclease, nuclease, CRISPR/Cas, His-Me endonuclease, recombinant endonuclease, and restriction endonuclease), which were more prevalent in pasture soils, particularly in PT1. These soils exhibited greater diversity and abundance, suggesting higher microbial diversity as well as a broader range of MGEs. Such patterns were also reported in fertilized soils (Liao et al. 2021), consistent with the findings presented in this study.

Endonucleases, proteins most prevalent in this study, represent one of the main defense strategies of bacteria to survive phages (Gao et al. 2020). One of the defense mechanisms of prokaryotes against phages using endonucleases is through the CRISPR-Cas system, more specifically CRISPR-Cas type VI Cas 13, which uses a single RNA subunit-guided Cas endonucleases to target and subsequently degrade exogenous RNA, generating adaptive immunity to the bacteria (Yang and Patel 2024). On the other hand, endonucleases are essential for phages in the formation of virions (Arens et al. 2019), which shows the diversity of functions for which endonucleases are used, both for phages and their hosts.

**Fig. 7:**
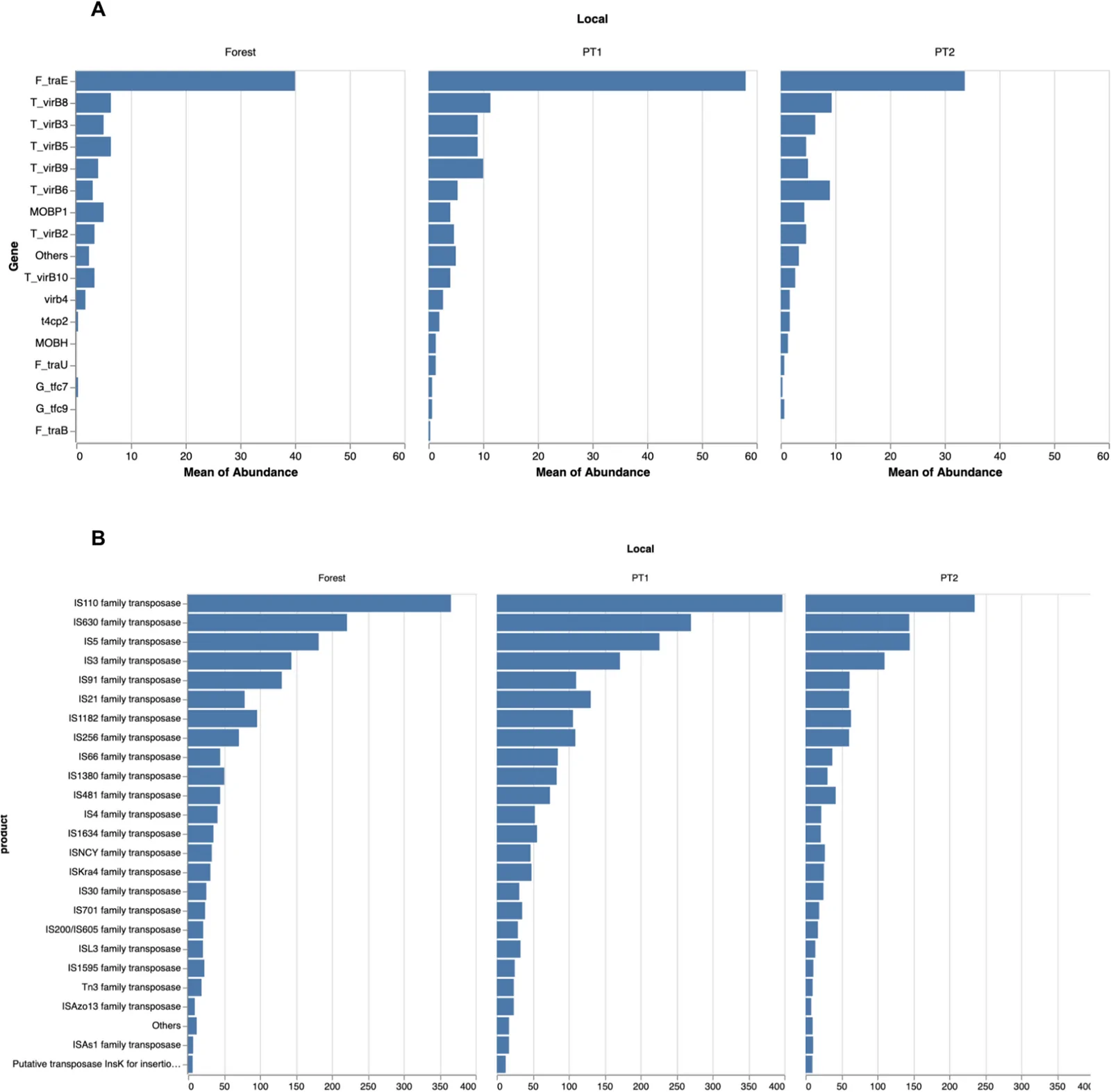
Abundances of different genetic components: (**A**) conjugable plasmids and (**B**) transposon families identified at the analyzed sites

In the case of the genetic components of conjugable plasmids, genes in the transfer region (*Tra*) of the F plasmid stand out, encoding the complex mechanism of the Type IV Secretion System (T4SS), responsible for bacterial conjugation (Costa et al. 2021). Among the different types of genes, the presence of the *traE* gene, involved in the formation of the final portion of the conjugative pilus (Virolle et al. 2020), is highlighted in all sites, but in relation to the previously analyzed genes, its abundance stands out in PT1 (Fig. 7A).

The main factor in the transmission of antimicrobial resistance is the transfer of resistance genes, which are frequently located on plasmids; that is, plasmid transmission by conjugation is one of the most important mechanisms for transmitting this type of resistance gene (Buckner et al. 2018; Jemounais et al. 2025). The study of this type of marker is important because it allows monitoring the spread of genes that are important for health.

Similarly, genes corresponding to the transposon family were identified, with IS10 genes standing out, followed by the IS630 and IS5 families, which are more prevalent at the PT1 site (Fig. 7B).

A decisive factor in the transmission of antimicrobial resistance is the conjugative transfer of resistance genes, which are frequently located on plasmids (Buckner et al. 2018; Jemouais et al. 2025). Bacterial conjugation, often referred to as bacterial sexual reproduction, is a key mechanism of horizontal gene transfer. In soils, it contributes to the rapid evolution and adaptation of bacterial strains by mediating the dissemination of diverse metabolic traits, including symbiotic lifestyles, virulence, biofilm formation, resistance to heavy metals, and, most notably, antibiotic resistance (Shan et al. 2022). These properties make conjugation a fundamentally important process and the focus of extensive research (Virolle et al. 2020), reinforcing the need to study plasmids. The *traE* gene, the most prevalent in this study, is homologous to *virB8* (the second most abundant gene detected), and encodes a membrane-associated protein that is an essential component of the type IV secretion system, which plays multiple roles, including conjugation. This protein can interact with *traD*, and such secretion systems are critical for bacterial survival and contribute significantly to virulence (Buckner et al. 2018; Casu et al. 2018). The presence of these genes may therefore be indicative of horizontal gene transfer via plasmids and is likely associated with bacterial persistence in the environment.

Genomic rearrangements, which include mutational genomic changes such as insertions, deletions, or inversions, are essential for genetic diversity (Durrant et al. 2024). In the case of transposase genes, these are ubiquitous in all domains of life (Vaysset et al. 2025). They are mobile elements that utilize transposition mechanisms, i.e., sequence insertion (SI) (Siddiquee et al. 2024), and are considered the simplest transposable elements, i.e., MGEs, found in prokaryotic genomes (Hiraizumi et al. 2024; Huang et al. 2023). The analysis of SI plays an important role in the adaptation of microbes to changes in the environment (Huang et al. 2023). Among the transposases, the most abundant in this study was that of the IS110 family. As from the IS110 family, these proteins are inserted into functional regions whose function is the translocation of As, Hg, Cu, Co, Zn, Cd, and sulfur oxidation (Huang et al. 2023), which indicates that such transposases may have a direct effect on nutrient cycling in the soil. At site PT1, the greater abundance of IS proteins was noteworthy. The excessive and indiscriminate use of fertilizers can cause an imbalance in the nitrogen cycle (Yoshiura et al. 2021), which could influence the abundance of this IS family.

Furthermore, the IS630 transposase stands out, representing the most widely distributed DNA superfamily in nature (Hu et al. 2024), as does the IS5 transposase, which is related to the response to environmental stress conditions (Onstead et al. 2024). According to Cardenas-Alegria et al. (2022), different soil characteristics, with heavy metals and other elements, can increase soil stress, which could favor the abundance of these mobile elements in the soil.

### Association of variables

**Fig. 8:**
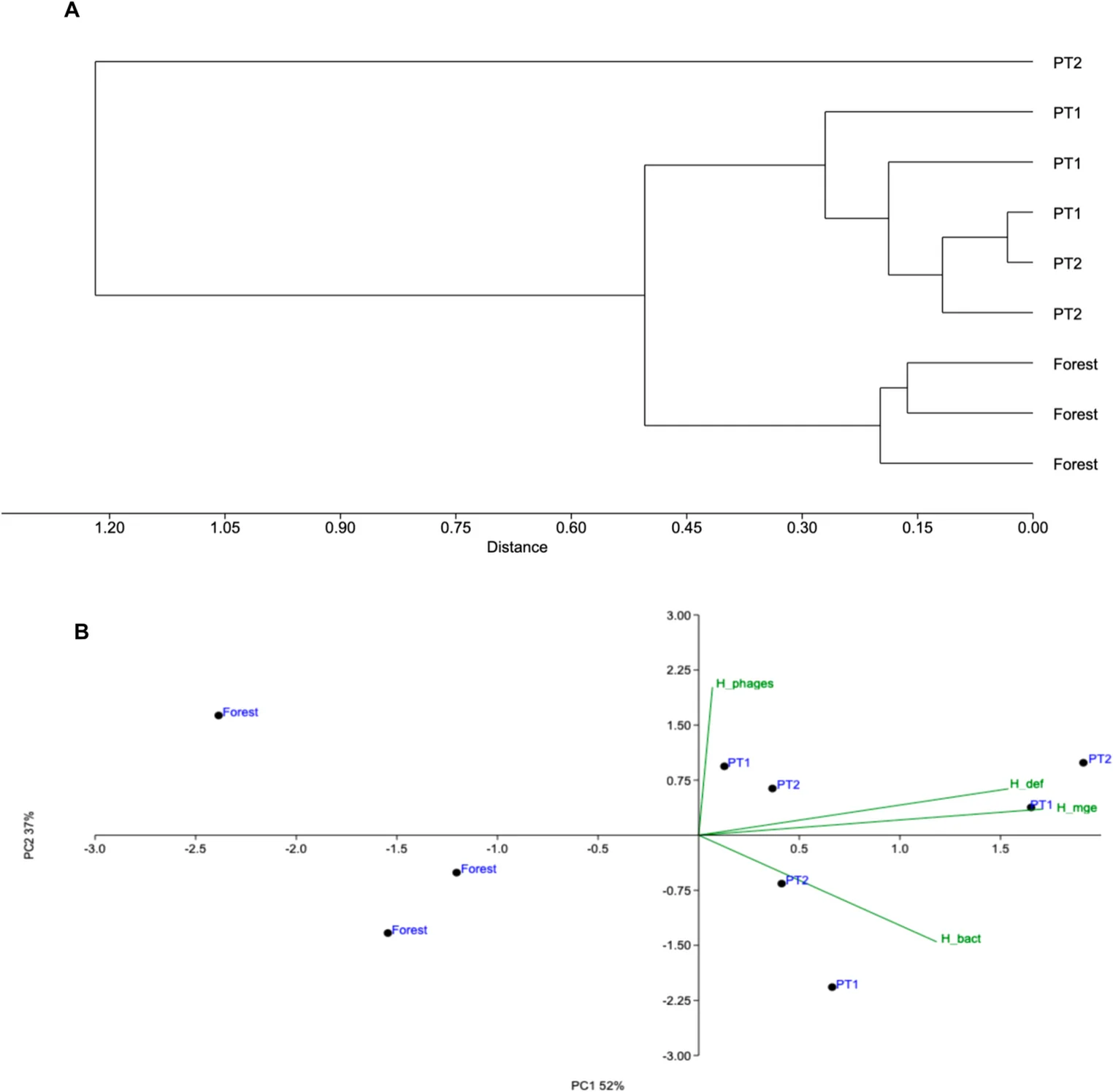
**A** Analysis of the clustering of Shannon alpha diversity indices of bacteria (H_bact), viruses (H_phages), defensome genes (H_def) and MGE (H_mge) and **B** principal component analysis

By analyzing the relationships between the alpha diversity indices of the bacteria, viruses, and defensoma and MGE genes, it was possible to show that the grouping of pasture sites presented similar characteristics, in contrast to the forest site, which presented different characteristics (Fig. 8A).

The difference between pasture sites and forest sites reinforces the idea that the effect of land use change to pasture has major impacts on soil characteristics, such as microbiota, diversity and abundance of microorganisms and genes (Sande et al. 2023; Tavares et al. 2025).

In the case of principal component analysis, the variability of the PC1 variables was above 52% (Fig. 8B), where it was possible to identify a correlation between the diversity indices of the defensome genes and the mobile elements (Pearson, *R* = 0.785; *p*-value = 0.012), (supplementary material Figure S4).

These changes in land use can affect the physical, chemical, and biological properties of the soil (Burton et al. 2022), impacting bacterial and archaeal communities (Venturini et al. 2022a, b). Thus, for example, the conversion of forest to pasture can alter the flow of methane gas in Amazonian soils, promoting a change in function and causing an accumulation in the atmosphere (Alvarez et al. 2023). The microbial change occurring in these soils shows a correlation between defensoma diversity and MGE, and the dynamics of the relationship between fungi and bacteria have been described (McDonnell et al. 2025).

The association between the diversity of MGE and defensome components stands out in contrast to other variables, as they are the main drivers of microbial evolution, enabling horizontal gene transmission and facilitating adaptation through the dissemination of relevant functional genes (Pacheco-Acosta et al. 2025). The transfer of these genes favors bacterial survival in the environment, which also suggests pressure from viruses on bacterial populations.

## Conclusions

Modified soils exhibited shifts in bacterial populations when comparing forest sites to pasture sites under two distinct treatments, indicating that pasture management can influence the dynamics of soil microbial communities. Moreover, a higher abundance of genes related to the defensome was detected in pasture soils compared to forest soils, suggesting that bacteria in these systems diversify their defense mechanisms against various processes of genetic transfer. Notably, genes associated with phages that infect enteric bacterial hosts (*E. coli* and *S. enterica*) were more abundant. In addition, a clear association between defensome gene diversity and mobile genetic elements was observed in pasture soils.

## Supplementary Information

Below is the link to the electronic supplementary material.

Supplementary Material 1

## Data Availability

Data availability statement: The datasets presented in this study can be found in online repositories. The names of the repository/repositories and the accession number(s) can be found at: www.ncbi.nlm.nih.gov/sra/PRJNA1214040.
