# Supplementary material for: Characterization of defensome genes and mobile genetic Elements in different types of pasture soil agroecosystems from the Brazilian Amazon
Source: Int Microbiol. 2026 May 19;29(7):1161–76. doi: 10.1007/s10123-026-00840-1 (PMC13653960; doi:10.1007/s10123-026-00840-1)
Supplement: Supplementary file 1 [file 10123_2026_840_MOESM1_ESM.docx]

**Suppementary material**

**Material Supplementary Figure S1.** Beta diversity analysis with Bray-Curtis distance, NMDS analysis of (**A**) bacteria, and (**B)** viruses

**Material Supplementary Figure S2.** Heatmap of relative abundance of the bacterial (**A**) and viral (**B**) families in soil samples from different sites. Native forest (F), pasture with treatment 1 (PT1), and pasture with treatment 2 (PT2)

**Material Supplementary Figure S3.** An abundance of elements related to endonucleases, terminases, polymerases, and other bacterial nuclear elements

**Supplementary material figure S4.** Pearson correlation analysis between Shannon alpha diversity indices of bacteria (H_bact), viruses (H_phages), defensome genes (H_def), and MGE (H_MGE)
